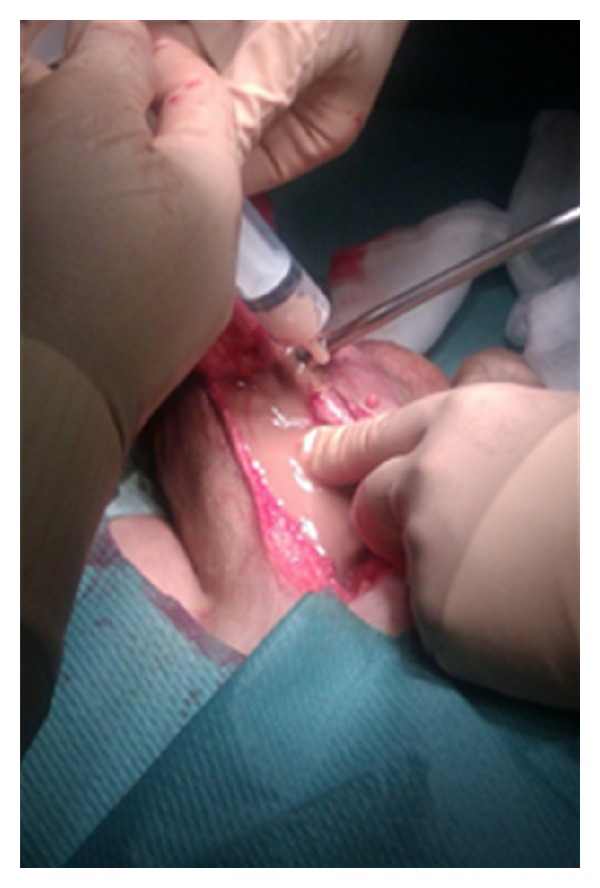# Intrascrotal Abscess, *Propionibacterium acnes* and *Staphylococcus cohnii* ssp. *cohnii*: A Case Report and Review of the Literature

**DOI:** 10.1155/2012/313694

**Published:** 2012-11-27

**Authors:** Masciovecchio Stefano, Alessandro Del Rosso, Pietro Saldutto, Giuseppe Paradiso Galatioto, Carlo Vicentini

**Affiliations:** Department of Life, Health, & Environmental Sciences, University of L'Aquila, “Giuseppe Mazzini” General Hospital, Teramo, Italy

## Abstract

*Introduction*. The *Propionibacterium acnes* and the *Staphylococcus cohnii ssp. cohnii* are occasional pathogenic bacteria. The intrascrotal localization of the *Propionibacterium acnes* is exceptional. The *Staphylococcus cohnii* ssp. *cohnii* is not able to colonize the urogenital apparatus but it is the most frequently responsible for blood culture contamination even if it can sustain, in particular conditions, systemic infections. *Case Presentation*. We report the case of a 72-year-old man who is under observation for pain and swelling of the left hemiscrotum associated to high fever. The scrotal ultrasound shows the presence of a left intra-scrotal abscess with didymus, epididymis, and intact didymus-epididymis tunicae. The blood culture executed for evening fever during antibiotic therapy has underlined an infection with *Propionibacterium acnes*. A following blood culture has shown an increase in *Staphylococcus cohnii* ssp. *cohnii*. Due to fever the patient has undergone left orchifunicolectomy with inguino-scrotal toilet. The anatomical pathological examination has also shown the presence of nonspecific granulomatous inflammation compatible with *Propionibacterium acnes* infection. *Conclusion*. The onset of an intrascrotal abscess likely sustained by *Propionibacterium acnes* complicated by a possible systemic *Staphylococcus cohnii* ssp. *cohnii* suprainfection is an exceptional event that, in our case, has been resolved with surgical toilet.

## 1. Introduction

Both the *Propionibacterium acnes (P. acnes) *and the *Staphylococcus cohnii ssp. cohnii (S. cohnii)* are bacteria that constitute part of the normal skin commensal flora and of the human mucous membranes that can, especially in particular conditions, behave like opportunistic pathogenic bacteria [1, 2]. The intra-scrotal infections are sustained exceptionally by *P. acnes. *The* S. cohnii, *according to the data present in literature, has not been related so far to infections of the uro-genital apparatus. We report a case of intra-scrotal abscess likely sustained by *P. acnes*, complicated by a probable systemic supra-infection due to *S. cohnii.*

## 2. Case Presentation

**Figure 1:**
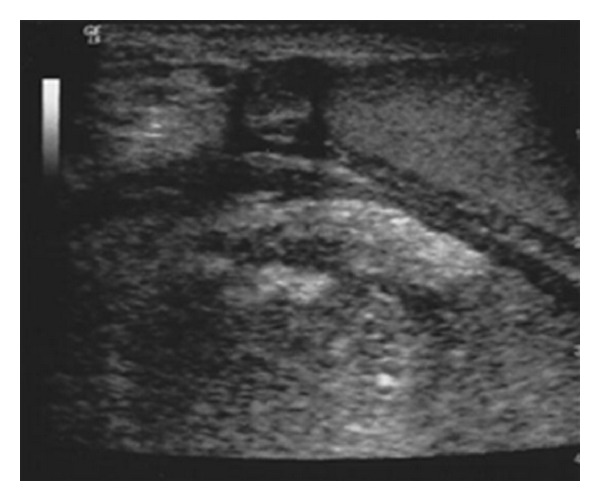


**Figure 2:**
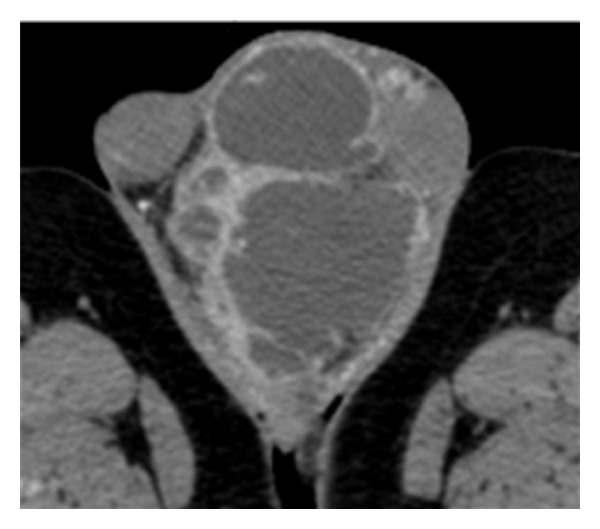


We report the case of a 72-year-old Caucasian man who is under observation for left hemiscrotal swelling, highly painful, associated to general malaise and high fever (39,2°C) arisen several days before. During the anamnesis he refers not to have been submitted to surgical interventions and to be affected by essential hypertension well controlled by drug therapy. Moreover, he refers to have taken an oral antibiotic therapy, about two months before, for 21 days (levofloxacina 500 mg/die) for a suspected left acute epididymitis with partial improvement of the personal case history but without complete resolution of the condition. During the inspection, the left emi-scrotus looks swollen and slightly iperemic while during the palpation the didymus appears easily appreciable, located within the anterolateral and proximal part but normal in form, dimension and consistency. We note, moreover, the presence of a scarsely floating taut-elastic consistency voluminous mass. The whole left hemiscrotum appears highly painfulon palpation. We execute a scrotal ultrasound that underlines a formation of 9 cm with uneven echostructure, prevalently hypoechoic, where we can recognize septatures with sporadic Doppler signals, which does not affect the testicular tunicae, the didymus and the ipsilateral epididymis. The bloodcount highlights exclusively a neutrophilic (18720/*μ*L −89,9%-) leukocytosis (20820/*μ*L). The other haematochemical examinations show an increase of the erythrocyte sedimentation rate (93 mm) and of the c-reactive protein (50,73 mg/mL) and a normality of emocoagulation, of the hydroelectrolithic balance and of the markers for the testicular cancer. Also the markers of renal and hepatic function are normal. The urine examination does not present abnormalities and the urine culture appears sterile. Showing the instrumental and laboratory case history an intra-scrotal abscess, we decide to start a medical poli-antibiotic empirical systemic therapy with imipenem 500 mg (1 fl × 3/die e.v.) and with teicoplanin 200 mg (1 fl/die i.m.) associated with a corticosteroid therapy with methylprednisolone 40 mg (1 fl × 2/die i.m.) and with a fluid-therapy. Three days after the beginning of the therapy we observe an improvement of the general conditions and an improvement of the local case history but we detect the onset of evening fever (maximum body temperature up to 37,6°C). So we decide to run a blood culture (3 blood collections) during every fever peak and to add to the treatment metronidazole 500 mg (1 fl × 3/e.v.). We analyse the report of the first blood culture examination executed 4 days after the blood collection that highlights a *P. acnes *infection in 2 of the 3 samples taken in absence of antibiogram. So we decide the suspension the ongoing therapy and to substitute it with meropenem 1 g (1 fl × 3/die e.v.), minocycline 100 mg (1 cpr/die) and rifampicin 300 mg (2 + 1 cpr × 2/die). The general conditions continue to improve, as opposed to the local conditions which remain stationary. We detect, moreover, the persistence of evening fever. In the twelfth day of hospitalization we run a blood culture that highlights, in only one blood collection, a* S. cohnii* infection highly sensitive to teicoplanin, rifampicin and fluoroquinolones. We decide the suspension exclusively of the therapy with minocycline and to substitute it with teicoplanin 400 mg (1 fl/die i.m.). After 5 days of treatment, because of the lack of improvement of the scrotal condition, demonstrated also during the echotomography (Figure 1), and of the fever (38,1°C), after the C.T. examination of the abdomen and pelvis (Figure 2), the patient is undergone left orchifunicolectomy with inguino-scrotal toilet and the resection of a little portion of scrotal skin (Figure 3). The anatomical-pathological examination of didymus and epididymis shows a substancial integrity of the structures while the removed para-testicular tissue shows large areas of fibrinoid necrosis, chronic and purulent acute inflammation, also lipophagic histiocytic and granulomatous gigantocellular, with newly formed connective tissue and areas of scleroialinosis. The cultural examination of the abscessual fluid has not highlighted bacterial growth. During the post operative course, which has been regular, repeted intra-scrotal washes with oxygen peroxide have been executed by drainage catheter. By the third post-operative day we observe a clear improvement of the general state with complete resolution of fever. At the discharge from hospital normal haematochemical examinations and excellent general conditions. At the control one month after the intervention excellent general conditions and complete healing of the surgical wound. 

## 3. Discussion

The *P. acnes *is a gram-positive pleiomorphic, rod-shaped, anaerobic, microaerophilic, aero-tolerant bacterium which forms part of the skin commensal microbial flora and mucous membranes. It is located mainly at the level of the pilosebaceous follicles and in the external auditory canal and also presents a particular tropism for conjunctiva mucous, oral cavity and intestines [3]. This bacterium can frequently act as opportunistic pathogen sustaining infectious-inflammatory processes in various organs and districts. The acne vulgaris is certainly the condition in which the correlation with *P. acnes* infection is better studied and known. More and more scientific evidences show how *P. acnes *can be associated with post-surgical infections especially in neurosurgery and ophthalmology, dentistry, orthopedics. It may also frequently colonize medical surgical devices [3]. This bacterium can be associated also with a variety of infective and inflammatory pathologies, like the endocarditis [4], the sarcoidosis [5] and the SAPHO syndrome (synovitis, acne, pustulosis, hyperostosis, osteitis) [5]. The *P. acnes* is not considered a uro-pathogen germ but a lot of studies underline a frequent localization of the prostate and a possible eziopatologic role in the prostate carcinoma [6]. A revision of the literature show how rare is the intra-scrotal colonization of the *P. acnes. *The only evidence present in literature is that reported by Yamamoto et al. that in 1991 have described the onset of an intra-scrotal and vesicular granuloma likely sustained by *P. acnes *[7]. This bacterium is sensitive to a large variety of antibiotics (*β*-lactam antibiotics, carbapenem, macrolides, glycopeptides and tetracyclines) [8], highly resistant to the therapy with metronidazol and weakly sensitive to the treatment with aminoglycosides [3]. Recent evidences show the sensitivity also to rifampicin [9]. The large use of the antibiotic therapy for the treatment of vulgar acne has shown an increase of the resistence incidence above all to the macrolides and to the tretracyclines [3, 8]. The* S. cohnii *is a gram positive stable coccus, coagulase negative and catalase positive, that behaves like a commensal muco-cutaneous bacterium. Revising literature we detect that the coagulase negative staphylocci are the principal germs responsible of the contamination of the collected blood [10]. The implantation of prosthetic material, the use of medical surgical devices, the immunodepression, the antibiotic broad spectrum poli-therapies and the prolonged hospitalization are factors which predispose the onset of *S. cohnii* systemic infections [11, 12]. Szymańska et al. have recently demonstrated as this germ is sensitive to glicopeptidic and aminiglicosid antibiotics, and also to rifamicines but above all to their combination [13]. From the revision of literature it emerges how the systemic infection sustained by *P. acnes* is a very rare event and that its intra-scrotal localization is a really exceptional condition. The empiric antibiotic therapy, in the impossibility to obtain an antibiogram, like in our case, should be based on the use of *β*-lactam antibiotics, carbapenems, glycopeptides, tetracyclines or rifamycins, better if combined. In our case the contextual resolution of the *P. acnes *infection and the demonstration of the *S. cohnii *infection can have a double interpretation. On one side this datum can be explained by a contamination of the blood sample because the coagulase negative staphylococci are the main contaminant of the blood cultural examination. On the other side it is probable that the prolonged antibiotic poli-therapy executed to eradicate the *P. acnes *infection has favoured the onset of a systemic *S. cohnii *supra-infection. Moreover, in our case, despite the prolonged broad spectrum antibiotic therapy, the lack of resolution of the local medical history and the recrudescence of the fever have addressed us to execute a demolition surgery. The absence of increase from the abscessual fluid, justified by the large use during the intervention of oxygen peroxide, that is a highly bactericidal agent for anaerobic germs and the presence, during the histology, of a granulomatous reaction are certainly consistent with a *P. acnes* infection [14]. 

## 4. Conclusion

The onset of an intra-scrotal abscess likely sustained by a usually non uro-pathogen bacterium like the *P. acnes *complicated by a possible *S. cohnii* systemic supra-infection is an exceptional event. The lack of resolution of the local case history or the worsening of the systemic conditions, despite a prolonged antibiotic poli-therapy, require an immediate surgical toilet that in our case has been completely resolutive.

## Figures and Tables

**Figure 3:**